# Distance and Cable Length Measurement System

**DOI:** 10.3390/s91210190

**Published:** 2009-12-16

**Authors:** Sergio Elias Hernández, Leopoldo Acosta, Jonay Toledo

**Affiliations:** 1 Department of Física Fundamental y Experimental Electrónica y Sistemas, Universidad de La Laguna, 38200, Spain; 2 Departament of Ingeniería de Sistemas Automática y Arquitectura y Tecnología de Computadores, Universidad de La Laguna, 38200, Spain; E-Mails: leo@isaatc.ull.es (L.A.); jonay@isaatc.ull.es (J.T.)

**Keywords:** distance, cable length, repetitive pulse, localization

## Abstract

A simple, economic and successful design for distance and cable length detection is presented. The measurement system is based on the continuous repetition of a pulse that endlessly travels along the distance to be detected. There is a pulse repeater at both ends of the distance or cable to be measured. The endless repetition of the pulse generates a frequency that varies almost inversely with the distance to be measured. The resolution and distance or cable length range could be adjusted by varying the repetition time delay introduced at both ends and the measurement time. With this design a distance can be measured with centimeter resolution using electronic system with microsecond resolution, simplifying classical time of flight designs which require electronics with picosecond resolution. This design was also applied to position measurement.

## 1. Introduction

Distance and location measurement systems have many important applications. Local positioning or localization can be achieved through two or more distance measurements. A mobile device, with local positioning techniques, can either gather the information about its position or can be localized from elsewhere. In this context, local-positioning systems attract significant attention [1].

The chosen technology depends on different circumstances such as outdoor or indoor use, range of distance to be measured, system costs, accuracy, *etc.* In most outdoor situations, where a clear line of sight to satellites is available, GPS is the chosen technology. Other available technologies under active research (some for indoor applications) are: sonar [2,3], time of flight [4], radio beacons [5], systems based on laser-optic technology [6,7], vision systems [8,9]. Table 1 shows a comparison of different measurement systems.

The prototype described in this paper is a low cost distance measurement system based on pulse repetition between a pair of transceivers. Under these conditions a frequency is generated by the endless travel of the pulse, this frequency is inversely proportional to the time of flight, so the distance between a pair of transceivers can be obtained. The main advantage of our system is that with microsecond electronics it is possible to measure distance with centimeter resolution. A traditional time of flight RF system needs picosecond electronic resolution to get similar distance resolution.

Other advantages of our system over other available and equivalent systems are that our system doesn't need direct vision from transmitters to receivers, as opposed to sonar or laser systems. Our system can be installed indoors, and our design is very cheap (Table 1).

## 2. System Description

**Figure 1.**
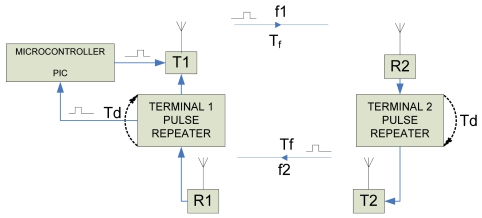
Block diagram of the prototype system.

As already mentioned, the proposed measurement system is based on the endless and continuous repetition of an electric pulse that propagates along the distance to be measured (Figure 1). At both ends of the distance or cable to be measured, there is pulse repetition hardware based on a monostable integrated circuit that generates a new pulse, after a constant delay, *T_d_*, every time a pulse is received, in reality *T_d_* will include all subsystem delays that are the delays introduced also by the transmitter and receiver. Supposing a zero distance is to be measured, the period *T* of every cycle will be. *T* = 2*T_d_*, because pulse repetitions introduce the same delay at both ends, *T_d_*. If the distance traveled by the wave is considered the new period will be, *T* = 2*T_d_* + 2*T_f_*, where *T_f_* is the time of flight, which is the time needed for the pulse to travel from terminal 1 to terminal 2 or vice versa, with *T* being the time a pulse needs to make a complete circle from terminal 1 to terminal 2 and back, see Figure1. If *d* is the distance to be measured and *v* the pulse wave propagation velocity, $T_{f}=\frac{d}{v}$, and $T=2\cdot (T_{d}+\frac{d}{v})$ being *T* the total time to make the circle: which, when expressed as frequency, *f*, implies:

(1) $$f=\frac{1}{T}=\frac{1}{2\cdot (T_{d}+\frac{d}{v})}$$

Equation (1) shows that the measured frequency *f* decreases as distance *d* increases.

The selection of the appropriate repetition delay on both terminals, *T_d_*, will fix the maximum and minimum frequencies captured for a range of distances. The sensitivity diminishes significantly as distance *d* increases, as can be seen in Equation (2) and Table 1:

(2) $$\frac{\partial f}{\partial d}=-\frac{1}{2v\cdot {\left(T_{d}+\frac{d}{v}\right)}^{2}}$$

**Figure 2.**
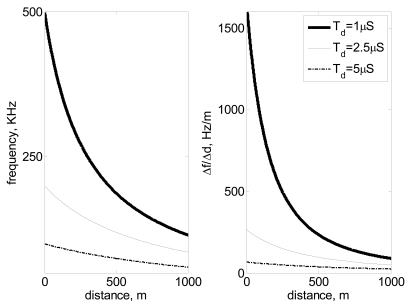
Frequency detected *versus* distance measured left, sensitivity as Δf/Δd *versus* distance right.

Equation (2) shows how the sensitivity $\frac{\partial f}{\partial d}$ varies significantly with distance d and pulse repetition delay, *T_d_*. One can observe that sensitivity significantly diminishes with both magnitudes. Figure 2 and Table 1, show how *f* and Δ*f* decrease nonlinearly with the distance *d* to be measured, following Equation (1) and Equation (2) respectively. The selection of an appropriate *T_d_* will depend on the range of distances to be measured and the precision, sensitivity and resolution required. Precision, sensitivity and resolution increase by reducing *T_d_*, but this reduction of *T_d_* has the drawback of increasing the bandwidth and the complexity of the electronic circuit.

**Figure 3.**
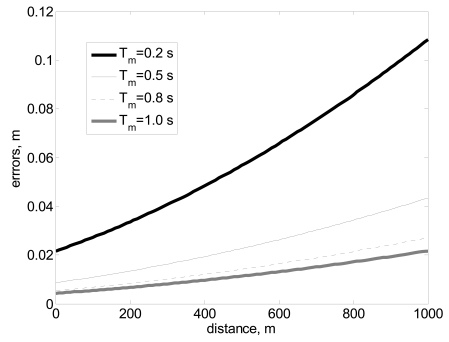
Distance *versus* errors as a function of measurement time *T_m_* in seconds.

The distance resolution depends also on measurement time. Distance calculation is defined by the frequency measurement, so the precision in the calculation of the frequency of the travelled pulsed is related to the distance resolution. Figure 3 shows the evolution of errors as a function of the measurement time. When the measurement time is high, the frequency is accurate and the error is low, if the measurement time is low, the error increases.

In our project, the main goal was to locate an autonomous vehicle over a 1 Km^2^ area with an accuracy of around 10 cm. In order to achieve this goal, *T_d_* = 2.68 *μS* is used on the prototype, this implies ${\text{f}}_{0}=\frac{1}{{\text{T}}_{\text{d}}}=186.6\text{KHz}$ and a sensitivity, Δ*f*/Δ*d*, ranging from 232 to 46 Hz (see Table 2). The measurement time was fixed at 1 second (*T_m_* = 1 s). With these parameters 1Hz resolution is obtained for the measured frequency with a theoretical accuracy in *d* between 1mm and 4 mm. The real accuracy will be lower due to variances in system parameters and RF bounces. As already mentioned, *T_d_* includes monostable repetition delays, and all other delays. The maximum distance to be measured was 1 Km, and the transmitters have a bigger range.

**Figure 4.**
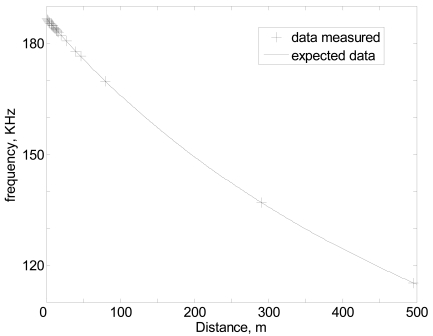
Frequency measured *versus* distance.

Real measured data can be observed in Figure 4, note that real data follows, with a high degree of accuracy, the predicted behavior shown in Equation (1).

## 3. Prototype Description

**Figure 5.**
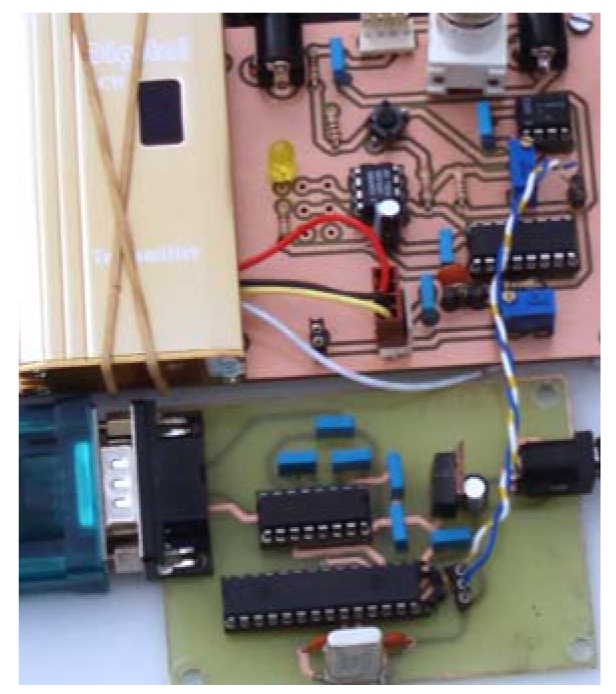
Terminal 1 Photograph, RF-transmitter Up-left, pulse repeater circuit up-right, and microcontroller circuit board bottom.

Our prototype as depicted in Figure 1 is composed of two different terminals, placed at both ends of the distance to be measured. Each terminal is composed of three different elements: 1. Radio frequency pulse transmitter (T1 and T2 in Figure 1), 2. Monostable Integrated Circuit, needed to repeat and delay the pulse (Pulse Repeater), and 3. Radio frequency pulse receiver, (R1 and R2). Apart from these elements Terminal 1 includes a simple microcontroller (Figure 5), whose tasks are:

- To generate the first pulse to be sent from terminal 1 to terminal 2.
- To detect the generated frequency using an inner microcontroller counter.
- To convert the captured frequency to distance based on Equation (1).

Our system uses two different frequencies to avoid interferences, one frequency f1 to send pulses from terminal 1 to terminal 2 and a second frequency, f2, for pulses from terminal 2 to terminal 1 (see Figure 1). It is obvious that the same system can be used to measure cable length, for this purpose each terminal should be located at each end of the cable, no radio module is needed because the electric pulse travels through the cable.

The material cost of our prototype, mainly integrated circuit cost, is less than 5 euros when used for cable length measurement and around 100 € when used for distance measurements, but in this last case, around 90% of this cost is for the radio data modules.

## 4. Cartesian Position of a Mobile System

**Figure 6.**
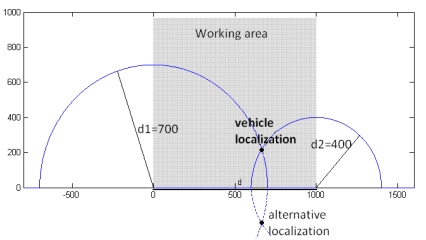
Localization schema, the distance between the two fixed points is known, and the prototype coordinates can be calculated using d1, and d2 distances.

The system described here can be used as a localization system. It's necessary for this application to measure two distances from two different fixed points (see Figure 6). Calculating the position of the vehicle is determined using these distances. Trilateration is applied in order to resolve the location problem. The localization scheme can be seen in Figure 6 the distance between the two fixed points (d), and between each one of these fixed points and the vehicle (d1,d2) are known, so the radius of the circumferences can be calculated, see Figure 6, and the (x,y) Cartesian position is resolved by equations (3) and (4).

When this schema with only two measurements (*d*_1_ and *d*_2_) is used, an alternative localization point appears. It's discarded because it is outside the working area. The system only works inside a predefined working area. The radius of each circle is the distance from each fixed point to theproto type. So Equation (3) is applied in order to calculate the vehicle localization (*x,y*):

(3) $$\begin{array}{l} d_{1}^{2}=x^{2}+y^{2}\\ d_{2}^{2}={\left(x-d\right)}^{2}+y^{2} \end{array}$$

Solving Equation (3) for x and y Equation (4) is obtained:

(4) $$\begin{array}{l} x=\frac{d_{1}^{2}-d_{2}^{2}+d^{2}}{2d}\\ y=\sqrt{d_{1}^{2}-x^{2}}=\sqrt{d_{1}^{2}-\frac{{\left(d^{2}+d_{1}^{2}-d_{2}^{2}\right)}^{2}}{4d^{2}}} \end{array}$$

**Figure 7.**
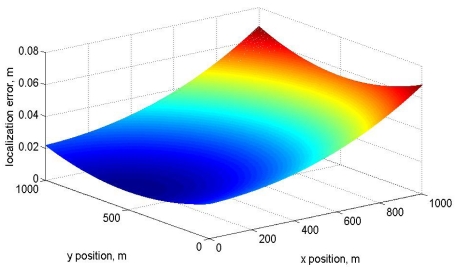
Localization error as a function of Cartesian positions.

Based on Equation (4) the system sensitivity, Equation (5), can be calculated for the Cartesian localization variables (*x,y*). The error is presented as a function of the Cartesian positions in Figure 7. It can be seen a minimum position error at the cartesian position *x* = 500, *y* = 0. This combination produces a minimum distance (*d*_1_ = 500, *d*_2_ = 500) to each fixed point. According to Equation (2) and Figure 3 the minimum error is reached when the distance is small and this is the lowest distance for each distance:

(5) $$\begin{array}{l} \Delta x=\left|\frac{\partial x}{\partial d_{1}}\right|\Delta d_{1}+\left|\frac{\partial x}{\partial d_{2}}\right|\Delta d_{2}\\ \frac{\partial x}{\partial d_{1}}=\frac{d_{1}}{d};\frac{\partial x}{\partial d_{2}}=-\frac{d_{2}}{d}\\ \Delta y=\left|\frac{\partial y}{\partial d_{1}}\right|\Delta d_{1}+\left|\frac{\partial y}{\partial d_{2}}\right|\Delta d_{2}\\ \frac{\partial y}{\partial d_{1}}=\frac{d_{1}\left(d^{2}-d_{1}^{2}+d_{2}^{2}\right)}{d^{2}\sqrt{-\frac{d^{4}+{\left(d_{1}^{2}-d_{2}^{2}\right)}^{2}-2d^{2}\left(d_{1}^{2}+d_{2}^{2}\right)}{d^{2}}}};\frac{\partial y}{\partial d_{2}}=\frac{d_{2}\left(d^{2}+d_{1}^{2}-d_{2}^{2}\right)}{2d^{2}\sqrt{d_{1}^{2}-\frac{{\left(d^{2}+d_{1}^{2}-d_{2}^{2}\right)}^{2}}{4d^{2}}}} \end{array}$$

The orientation of the prototype can be obtained if two localization systems are installed on the mobile device. These systems must be separated to get the Cartesian coordinates of each system (*x*_1_,*y*_1_), (*x*_2_,*y*_2_). The prototype orientation can be calculated with these coordinates using Equation (6) where *x*_dif_ is the difference between *x*_1_ and *x*_2_ coordinate and *y*_dif_ between *y*_1_ and *y*_2_. The error in the orientation is proportional to the distance of the systems installed in the prototype and the precision in the (*x,y*) calculus:

(6) $$\begin{array}{l} \alpha =\arctan\left(\frac{y_{1}-y_{2}}{x_{1}-x_{2}}\right)=\arctan\left(\frac{y_{\text{dif}}}{x_{\text{dif}}}\right)\\ \Delta \alpha =\left|\frac{\partial \alpha }{\partial x_{1}}\right|\Delta x_{1}+\left|\frac{\partial \alpha }{\partial x_{2}}\right|\Delta x_{2}+\left|\frac{\partial \alpha }{\partial y_{1}}\right|\Delta y_{1}+\left|\frac{\partial \alpha }{\partial y_{2}}\right|\Delta y_{2}\\ \frac{\partial \alpha }{\partial x_{1}}=\frac{-y_{\text{dif}}}{x_{\text{dif}}^{2}\left(1+\frac{y_{\text{dif}}^{2}}{x_{\text{dif}}^{2}}\right)};\frac{\partial \alpha }{\partial x_{2}}=\frac{y_{\text{dif}}}{x_{\text{dif}}^{2}\left(1+\frac{y_{\text{dif}}^{2}}{x_{\text{dif}}^{2}}\right)}\\ \frac{\partial \alpha }{\partial y_{1}}=\frac{1}{x_{\text{dif}}\left(1+\frac{y_{\text{dif}}^{2}}{x_{\text{dif}}^{2}}\right)};\frac{\partial \alpha }{\partial y_{2}}=\frac{-1}{x_{\text{dif}}\left(1+\frac{y_{\text{dif}}^{2}}{x_{\text{dif}}^{2}}\right)} \end{array}$$

**Figure 8.**
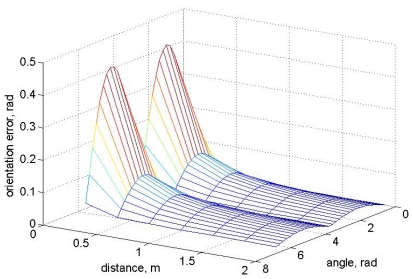
Orientation error angle as a function of distance between sensors and orientation angle for a fixed Cartesian position.

**Figure 9.**
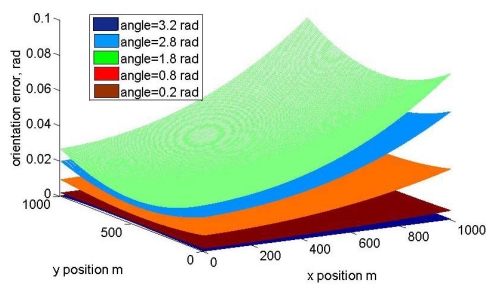
Orientation error angle as a function of Cartesian position for a distance between sensors of 1 m, each graph represents a different orientation.

Figure 8 shows the orientation error as a function of the distance between the sensors and orientation angle. The cartesian position of the vehicle is fixed to *x* = 500 m, *y* = 500 m in this graph. If the error in Cartesian position is represented, Figure 9 is obtained, where the distance between the two sensors in the prototype is fixed at 1 m. The error as a function of the orientation is plotted with different colors.

## 5. Non Static Measurements

**Figure 10.**
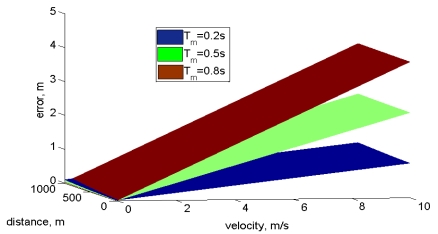
Distance calculus error as a function of prototype travel velocity with different measurement times.

If the prototype is moving, the frequency measurement changes as a function of the vehicle velocity *V_ν_*, and measurement time *T_m_* Equation (7). The error increases with the prototype speed and with the measurement time Figure 10. For a mobile system better precision is obtained if small measurement times are used. Error will be proportional to *V_ν_* and *T_m_* so the main error source is proportional to the prototype speed movement. Figure 10 shows the evolution of error as a function of speed and distance for different measurement times. The main error source is velocity, so in order to get a better distance estimation it's necessary to reduce the measurement time. A detail is presented in Figure 10 where the nonlinear error as a function of distance can be appreciated:

(7) $$f=\frac{1}{T}=\frac{1}{2\cdot (T_{d}+\frac{d}{v})}\Rightarrow f=\frac{1}{2\cdot \left(T_{d}+\frac{d_{0}+\frac{1}{Tm}\int_{0}^{Tm}tV_{v}\partial Tt}{v}\right)}=\frac{1}{2\cdot \left(T_{d}+\frac{d_{0}+\frac{Tm}{2}V_{v}}{v}\right)}$$

Another way to see the error is as a measurement delay. Position is not got in real time, the position is obtained $\frac{TmV_{v}}{2}$ seconds before, when the prototype was located in a previous place. When the system stops, the position will be calculated again with good resolution.

## 6. Conclusions

A very simple prototype for distance and cable length measurements is built based on a simple algorithm. The basis of our device is continuous repetitions of an electric pulse that endlessly travels backwards and forwards through the distance or cable to be measured. The endless travel of the pulse generates a frequency that varies almost inversely with the distance to be measured. The frequency range and resolution of the distance measured will depend on the range of the distances to be measured, and the time delay it takes for the pulse to be repeated at each end. The proposed system uses a simple and low cost electronic device based on a basic microcontroller that performs the main tasks. The system has been successfully tested for distances from 0 to 500 m, with 10 cm accuracy. This system can be used as a local position system to get position and orientation of mobile systems.

## Figures and Tables

**Table 1.** Comparison with other distance measurement systems.

**Technology**	**Resolution**	**Price**	**Distance**	**Details**
Ultrasound	Medium, 3–5 cm	Low	Low, 8 meters	Very low distance Needs direct vision Simple Electonics
Laser	High, 2–5 mm	High	High, Km	Needs direct vision Picosecond electronics
Vision	Medium, 10 cm	Low	Low, 20 meters	Computer processing Landmarks installed in the field
Radar	Low, 30 cm	High	High, Km	Picosecond electronics
DGPS	High, 10 cm	Medium	High, Km	Only outdoor use

**Table 2.** Frequencies and sensitivity *versus* distances: *T_d_* (μS) time delay for pulse repetition, *f*_0_ and *f*_1Km_ frequency detected for 0 or 1 Km distance, Δ*f*_10_/Δ*d* and Δ*f*_1Km_/Δ*d* sensitivity at 10 m and 1 Km respectively.

***T_d_* (μS)**	***f*_0_ (KHz)**	***f_1Km_* (KHz)**	**Δ*f*_0_/Δ*d*** **(Hz/m)**	**Δ*f*_10_/Δ*d*** **(Hz/m)**	**Δ*f*_100_/Δ*d*** **(Hz/m)**	**Δ*f*_1Km_/Δ*d*** **(Hz/m)**
0,1	5,000	145.8	161.3 × 10^3^	96154	8,944.5	141.5
1	500	115.5	1661.1	1565.9	939.85	88.8
**2.68**	**186.6**	**83.1**	**232.0**	**226.3**	**183.5**	**46.1**
10	50	37.5	16.66	16.562	15.614	9.38
100	5	4.84	0.167	0.16656	0.16557	0.156
1,000	0.5	0.498	1.67 × 10^−3^	1.66 × 10^−3^	1.66 × 10^−3^	1.65 × 10^−3^

## References

[1] Hightower J., Borriello G. (2001). Locations systems for ubiquitous computing. IEEE Comput..

[2] Tards D., Neira J., Newman P.M., Leonard J.J. (2002). Robust mapping and localization in indoor environments using sonar data. Int. J. Robot. Res..

[3] Navarro D., Benet G., Martinez M. (2007). Line based robot localization using a rotary sonar. IEEE Conf. Emerg. Tech. Factory Autom..

[4] Larsson U., Forsberg J., Wernersson A. (1996). Mobile robot localization: Integrating measurements from a time-of-flight laser. IEEE Trans. Ind. Electron..

[5] Djugash J., Singh S., Corke P.I. (2005). Further results with localization and mapping using range from radio. Int. Conf. Field Serv. Robot..

[6] Zhou Y., Lu W., Huang P. Laser-activated RFID-based Indoor Localization System for Mobile Robots.

[7] Hernández S.E., Torres J.M., Morales C.A., Acosta L. (2003). A new low cost system for autonomous robot heading and position localization in a closed area. Auton. Robots.

[8] Agrawal M., Konolige K., Bolles R.C. (2005). Robust vision-based localization by combining an image-retrieval system with Monte Carlo localization. IEEE Trans. Robot..

[9] Menegatti E., Pretto A., Scarpa A., Pagello E. (2006). Omnidirectional vision scan matching for robot localization in dynamic environments. IEEE Trans. Robot..

